# BK_Ca_ Channels as Targets for Cardioprotection

**DOI:** 10.3390/antiox9080760

**Published:** 2020-08-17

**Authors:** Kalina Szteyn, Harpreet Singh

**Affiliations:** Deptartment of Physiology and Cell Biology, The Ohio State University, Columbus, OH 43210, USA; Kalina.szteyn@osumc.edu

**Keywords:** potassium channels, acute myocardial infarction, cardioprotection, BK_Ca_ channels, ischemia-perfusion injury, reactive oxygen species, mitochondria

## Abstract

The large-conductance calcium- and voltage-activated K^+^ channel (BK_Ca_) are encoded by the *Kcnma1* gene. They are ubiquitously expressed in neuronal, smooth muscle, astrocytes, and neuroendocrine cells where they are known to play an important role in physiological and pathological processes. They are usually localized to the plasma membrane of the majority of the cells with an exception of adult cardiomyocytes, where BK_Ca_ is known to localize to mitochondria. BK_Ca_ channels couple calcium and voltage responses in the cell, which places them as unique targets for a rapid physiological response. The expression and activity of BK_Ca_ have been linked to several cardiovascular, muscular, and neurological defects, making them a key therapeutic target. Specifically in the heart muscle, pharmacological and genetic activation of BK_Ca_ channels protect the heart from ischemia-reperfusion injury and also facilitate cardioprotection rendered by ischemic preconditioning. The mechanism involved in cardioprotection is assigned to the modulation of mitochondrial functions, such as regulation of mitochondrial calcium, reactive oxygen species, and membrane potential. Here, we review the progress made on BK_Ca_ channels and cardioprotection and explore their potential roles as therapeutic targets for preventing acute myocardial infarction.

## 1. Introduction

In the early 1980s, ‘big K’ channel (BK_Ca_), named after its large single-channel conductance 250–300 pS (in symmetrical 150 mM KCl), was originally cloned in *Drosophila* at the slowpoke (slo) [1,2]. In mammals, BK_Ca_ channels were defined as large-conductance calcium-activated potassium ion channels (BK_Ca_, MaxiK, K_Ca_1.1, Slo1) and were characterized in bovine chromaffin cells, rabbit skeletal muscle, and rat muscle cells [1,2,3]. BK_Ca_ channels are ubiquitously expressed in a broad range of excitable and inexcitable cell types [4], as well as in intracellular organelles, including mitochondria [5], nuclei [6], endoplasmic reticulum [7,8], and lysosomes [2,9,10], where they are termed as iBK_Ca_ [10]. BK_Ca_ channels participate in a wide variety of fundamental physiological processes from vascular tone [11,12], cardiac rhythmicity [11,13], erectile and urinary autonomic functions [14,15], regulation of gene expression [6], and aging [16]. Recent reports suggest that alterations of BK_Ca_ function and expression may associate with several pathological conditions, such as paroxysmal nonkinesigenic dyskinesia with a gain of function, and cerebellar ataxia with loss-of-function mutations [17,18], making them unique therapeutic targets. The exclusive initiative by the ‘*KCNMA1 channelopathy international advocacy foundation*’ has brought BK_Ca_-associated disorders to the forefront and facilitates education, treatment, and networking opportunities for KCNMA1 channelopathy patients, physicians, and researchers [19].

The activity of BK_Ca_ channels is regulated by two physiological stimuli: Membrane depolarization and intracellular calcium levels, and either calcium or voltage can independently modulate its function [20,21]. Under normal physiological conditions, both factors simultaneously modulate BK_Ca_-mediated ion currents, but in the absence of calcium, membrane depolarization alone can elicit BK_Ca_ currents. On the other hand, calcium binding decreases the energy required to open the channel and causes a shift of open probability (P_O_) towards more negative voltages [21,22]. *Kcnma1* gene codes for α-subunit that assembles into a pore-forming tetramer, a primary BK_Ca_ channel functional unit [23]. α-subunit consists of the transmembrane region (S0-S6) and large intracellular C-terminus [23]. S1-S4 transmembrane segments create the voltage-sensing domain, whereas S5-S6 form the channel pore. The C-terminus contains ligand-binding motifs, phosphorylation sites, and a stretch of negatively charged amino acids that form ‘Ca^2+^ bowls’ [24]. In addition to pore-forming, α-subunit tetramer can co-assemble with auxiliary subunits, β (β1- β4) and γ (γ1- γ4) [24,25,26,27]. The channel pore can co-assemble with zero to four regulatory subunits, and the number and kind of regulatory subunits present can significantly alter channel performance as well as its activity [27]. BK_Ca_ channels with different subunit combinations can present a wide range of channel activity, rates of activation or deactivation, current rectification properties, and sensitivity to pharmacological agents [27]. When taking into consideration the number of splice variants and a plethora of combinations in which BK_Ca_ channels can assemble, one can understand the functional diversity that is uniquely calibrated for the needs of a variety of cells in which they are expressed [4,5,10,17].

Though BK_Ca_ channels are uniformly present in all cell types, they have recently been brought into the forefront in cardiovascular studies [4,10,28,29,30,31,32,33,34]. They have been implicated in several pathophysiological conditions and cardiovascular diseases [2,5,35,36,37,38,39,40,41,42,43,44,45,46,47,48,49,50,51]. In this review, we exclusively focus on the functions of BK_Ca_ channels in the cardiac system and their role in protection from acute myocardial infarction (AMI).

## 2. Localization of BK_Ca_ Channels

BK_Ca_ channels are heterogeneously expressed in the cardiovascular system. BK_Ca_ channels are present in the plasma membrane of vascular smooth muscle, where they contribute to the regulation of resting membrane potential [52,53] and play a critical role in myogenic tone regulation [48,49,54]. BK_Ca_ channel activity provides hyperpolarizing currents that facilitate negative feedback to vasoconstriction induced by high intravascular pressure [48]. BK_Ca_-dependent vasoregulation in smooth muscles is attributed to the coupling of α subunits with its regulatory β1 subunit [48]. This configuration of the channel increases its Ca^2+^ sensitivity, changes gating energetics, and current kinetics, therefore, shifts its open probability towards more negative voltages than those determined for channels formed by α subunits alone [25,48,55]. Furthermore, β1 knockout mice showed decreased BK_Ca_ calcium sensitivity, which leads to increased arterial tone and blood pressure [48,56].

BK_Ca_ also plays an active role in heart rate regulation. In 2010, the first evidence was presented for the presence of BK_Ca_ channels in the heart and its involvement in the regulation of heart rate [57]. Heart rate is determined by the sinoatrial node (SAN), which is a primary pacemaker of the cardiac conduction system [58]. Electrophysiological characterization of isolated SAN cells showed a 55 ± 15% reduction in action potential (AP) firing when paxilline (BK_Ca_ specific inhibitor) was applied [11]. Moreover, SAN cells isolated from *Kcnma1^-/-^* mice showed a 33% lower baseline firing rates when compared to wild type [11]. These findings indicate the presence of BK_Ca_ channels in the plasma membrane of SAN cells. BK_Ca_ in SAN are shown to be functionally coupled with L-type calcium channel Ca_v_1.3, which are expressed in sinoatrial and atrioventricular nodes but not in ventricular cardiomyocytes, and are responsible for pacemaker currents [59]. Both channels are located at the plasma membrane within the “Ca^2+^ nanodomain” region, and influx through Ca_v_1.3 guarantees a local Ca^2+^ increase sufficient enough for BK_Ca_ activation at physiological voltages [60].

**Figure 1:**
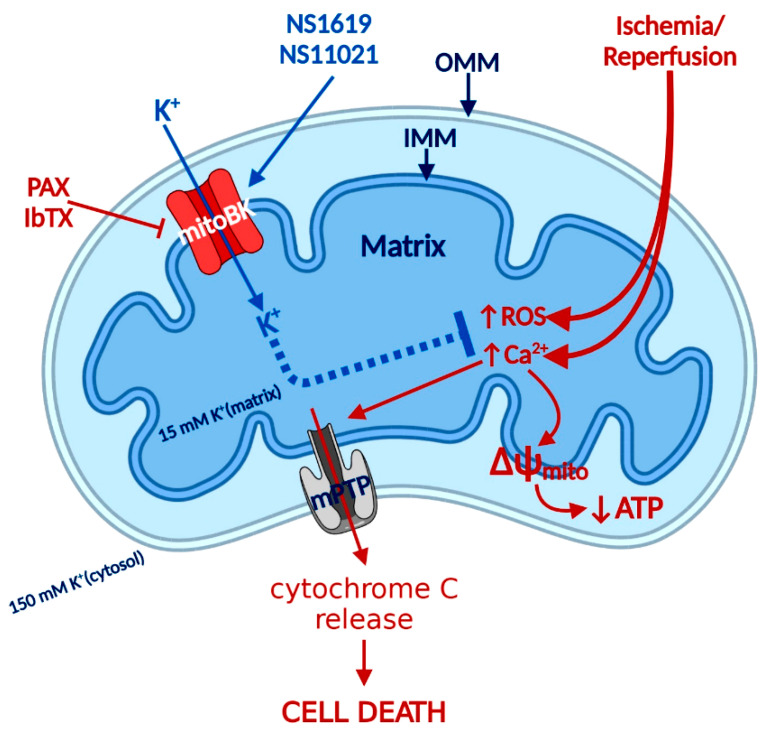
BK_Ca_ signaling in cardiomyocytes mitochondria during ischemia-reperfusion (I/R) injury. I/R injury causes an increase of reactive oxygen species (ROS) production and Ca^2+^ overload that leads to mPTP opening, the collapse of membrane potential (ΔΨ_IMM_), and the release of cytochrome C that causes cell death. The opening of BK_Ca_ protects the heart by reducing ROS and increasing the calcium retention capacity, hence delaying the opening of mPTP. IMM—Inner mitochondria membrane, OMM—Outer mitochondria membrane. mitoBK_Ca_ activators (NS1619, NS11021), mitoBK_Ca_ inhibitors (PAX-paxilline, IbTX-iberiotoxin), mPTP-mitochondrial permeability transition pore.

BK_Ca_ channels have not been detected in the plasma membrane of adult cardiomyocytes; on the other hand, comprehensive electrophysiological, biochemical, and pharmacological data indicated the presence of BK_Ca_ channels in the inner mitochondrial membrane (IMM) of cardiomyocytes (Figure 1) [5,38,43]. Mitochondrial BK_Ca_ (mitoBK_Ca_) is a product of the *Kcnma1* gene, the same gene that encodes the α subunit of the plasma membrane BK_Ca_ channel, but in cardiomyocytes, a specific DEC splice variant determines protein mitochondrial localization [5]. mitoBK_Ca_ channel activity increases K^+^ conductance and improves mitochondria respiratory function by reducing the production of reactive oxygen species (ROS) and decreasing deleterious intra-mitochondrial Ca^2+^ accumulation, which plays a major role in heart protection against ischemia/reperfusion (I/R) injury [5,34,46,61,62]. Surprisingly, BK_Ca_ channel currents were measured on the cell membrane of ventricular myocytes isolated from chick embryos [63,64,65,66]. Currents measured in chick embryonic ventricular myocytes showed all the hallmark characteristics of BK_Ca_ channels, including iberiotoxin sensitivity and “big” single-channel conductance of approximately 270 pS [64,66]. The absence of BK_Ca_ channels in the cell membrane of adult cardiomyocytes but the presence in embryonic cardiomyocytes strongly suggests that during development, BK_Ca_ channels undergo a change of the expressed splicing variant that determines mitochondrial but not plasma membrane localization. Further investigations will be needed to probe the expression pattern of the BK_Ca_ channel during cardiac developmental stages.

Fibroblasts constitute a significant fraction of the heart and they are key determinants of both the structure and function of the myocardium [67]. Functional expression of BK_Ca_ channels was identified in cultured human cardiac fibroblasts [68,69,70,71]. Potassium currents were sensitive to highly selective BK_Ca_ inhibitors, paxilline, and iberiotoxin and demonstrated 162 ± 8 pS single-channel conductance [69]. Cardiac fibroblasts play a central role in the maintenance of the extracellular matrix in normal hearts and act as mediators of inflammatory and fibrotic myocardial remodeling in the injured heart. It is also becoming increasingly clear that cardiac fibroblasts contribute to the electrophysiological remodeling mediated by BK_Ca_ channels [69]. One of the mathematical simulations predicts that BK_Ca_ channels activity in cardiac fibroblasts may contribute to the alteration in myocardial action potential when tight electrical coupling forms between those two types of cells [69]. Furthermore, fibroblasts may act as a current “sink” and prevent impulse propagation that leads to the development of cardiac arrhythmias, but the role of BK_Ca_ in this process needs further evaluation to decipher the precise role and mechanism involved [72].

## 3. Role of BK_Ca_ Channel Agonists

**Figure 2:**
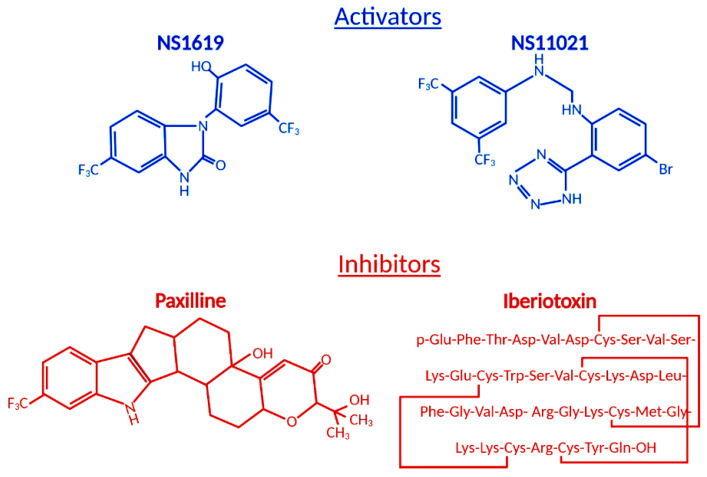
Structural formulas of commonly used mitochondrial BK_Ca_ modulators.

The ubiquitous BK_Ca_ expression, a dual activation mechanism that allows them to couple intracellular signaling to membrane potential and significantly modulate physiological responses in a plethora of tissues, prompted intense development of BK_Ca_ channel modulators [73,74]. The number of identified molecules is significant and growing, which is why we will limit this review to regularly used pharmacological tools (Figure 2) to study BK_Ca_.

Among the first synthetic BK_Ca_ activators were benzimidazoles NS004 [75] and NS1619 [76] (Figure 2) and the latter became one of the most used agonists in establishing the physiological functions of BK_Ca_. NS1619 accelerated K^+^ mitochondrial uptake, improving mitochondrial respiratory function, but its link to cardioprotection was made when NS1619 was administered prior to the ischemic event and protected isolated perfused rat hearts from global I/R injury [38]. Subsequent studies confirmed that the administration of NS1619 protected the heart from I/R injury in mice [77,78], rats [79,80], guinea pigs [41], rabbits [43], and dogs [81]. To further elucidate cardioprotective mechanisms, several studies probed ROS production and mitochondrial Ca^2+^ levels during ischemia and reperfusion. Isolated guinea pig hearts were subjected to I/R injury in the presence and absence of NS1619 [41]. Hearts were nearly continuously monitored for levels of nicotinamide adenine dinucleotide (NAD) + hydrogen (H) (NADH), superoxide, and mitochondrial calcium and NS1619 showed attenuated levels when compared with untreated hearts that resulted in an astounding 50% decrease in infarct size [41]. Those effects were nullified by paxilline and superoxide dismutase, showing that both BK_Ca_ channel activity and superoxide are necessary for the cardioprotective effect [41]. However, an increased number of investigations showed poor potency and inadequate selectivity of NS1619. At higher concentrations, NS1619 (at ~100 µM) inhibits L-type Ca^2+^ channels in rat ventricular cardiomyocytes [82], Ca^2+^-activated chloride channels [83], and voltage-activated Ca^2+^, K^+^, and Na^+^ channels [76,84,85]. Additionally, few studies determined non-ion channel effects of NS1619 on functions of other proteins like endoplasmic reticulum Ca^2+^-ATPase and complex I, ATP-synthase, which raised questions about the role of BK_Ca_ channels in cardioprotection [86,87]. Our group clarified pharmacological controversy by showing cardioprotective effects of NS1619 (up to 30 µM) in wild-type mice that were not observed in *Kcnma1^-/-^* animals, additionally showing that pretreatment with NS1619 increases the mitochondrial Ca^2+^ retention capacity in wild-type but not in *Kcnma1^-/-^* animals [5]. Because of the limited usefulness of NS1619, the need for a specific and sensitive activator led to the development of NS11021. NS11021 displayed better specificity and potency (10 times higher than NS1619) and activation of BK_Ca_ by parallel shifting the channel activation curves to more negative potentials [88]. The single-channel analysis revealed that NS11021 increased the open probability by altering the gating kinetics without altering the single-channel conductance [88]. It also did not influence the number of cloned K_V_ channels or endogenous Na^+^ and Ca^2+^ channels in cardiomyocytes isolated from guinea pigs [88]. In nanomolar concentrations, NS11021 displayed beneficial effects on mitochondria by accelerating the initial K^+^ uptake by 2.5 fold, an increase in mitochondrial volume with very little effect on membrane potential, which translates into an increase in the mitochondrial respiratory response [61]. Studies have also shown an improvement of mitochondrial energy production via oxidative phosphorylation, leading to increased ATP sustainability, and allowing cardiomyocytes to tolerate greater oxygen deprivation [42,61]. This leads to reduced mitochondrial Ca^2+^, delayed opening of mitochondrial permeability transition pore (mPTP), postponement of apoptosis initiation, and ultimately, mitoBK_Ca_ channel activity prolongs the survival of cardiomyocyte during ischemic insult [41,89].

In addition to previously mentioned synthetic activators, a plethora of endogenous modulators have been studied that induce similar changes in BK_Ca._ These molecules include gasotransmitters, such as nitric oxide (NO), carbon monoxide (CO), and hydrogen sulfide (H_2_S) [90]. CO has been shown to increase the open probability (P_O_) of BK_Ca_ by mimicking the action of Ca^2+^ because the mutation of residues responsible for Ca^2+^ sensitivity also prevented channel CO sensitivity [91,92]. Furthermore, the same mechanism appears to be responsible for BK_Ca_ activation by H^+^, which could contribute to BK_Ca_’s cardioprotective function, as intracellular pH falls by 0.5 to 1 unit during the early stages of ischemia and changes in extracellular acidosis had no significant effect on BK_Ca_ [92,93,94,95,96]. NO is a well-established vasodilatory factor released by endothelial cells and it has been shown to significantly increase P_O_ of BK_Ca_ by direct interactions and/or through cyclic guanosine monophosphate (cGMP)-mediated phosphorylation [97,98]. Another molecule from the gasotransmitter group is H_2_S, a biologically active gas that plays a role in the physiology and pathophysiology of cardiovascular and nervous systems [99,100]. There are conflicting data showing contradictory results that vary from inhibition of native and recombinant currents of the α subunit expressed in human embryonic kidney (HEK)-293 cells [101], through to β1 subunit presence and enhanced oxidative regulation and BK_Ca_ activation [102], to the BK_Ca_ response dependent on channel priming by PKG phosphorylation [103]. These findings indicate the complexity of BK_Ca_ signaling, which heavily depends on allosteric interactions, oxidative state, phosphorylation, divalent ion concentration, and voltage. MitoBK_Ca_, because of their localization, are continuously exposed to ROS. It has been reported that H_2_O_2_ increased the survival of cardiomyocytes that underwent I/R simulation [104]. However, BK_Ca_ regulation by ROS depends on oxidative species, which can activate, inhibit, or leave the channel unaltered [90,105]. Data suggest that superoxide anion (O_2_^−^) has little effects on BK_Ca_ function, peroxynitrite (ONOO^−^) decreases activity, and H_2_O_2_ action depends on the tissue type and experimental conditions and it may have both an excitatory and inhibitory effect [90,105].

## 4. Role of BK_Ca_ Channel Antagonists

Antagonists and inhibitors (Figure 2) of BK_Ca_ channels are widely used for cardioprotection studies. One of the most widely used small molecules and synthetically derived BK_Ca_ channel blockers is tetraethylammonium chloride (TEA) [106,107]. TEA blocks channel activity by binding within the ion conduction pathway in a voltage-dependent manner from both sides of the membrane; however, it lacks selectivity for BK_Ca_ channels as TEA blocks several voltage-gated potassium channels [108,109]. Venom from scorpions is an invaluable source of BK_Ca_ inhibitors. The first potent BK_Ca_ blocker was a 37-amino-acid peptide charybdotoxin (ChTX) identified in 1985 [110]; however, because of its inhibitory effect on K_v_1.3 and intermediate-conductance Ca^2+^-activated-K^+^ channels (IK channels), currently, ChTX use in BK_Ca_-specific studies is limited [111]. Another 37-aa peptide isolated from scorpion venom was iberiotoxin (IbTX), which showed high selectivity and affinity for BK_Ca_ channels [112]. The blocking mechanism of ChTX and IbTX is attributed to their binding to the external pore region of the channel through bimolecular reaction and physically blocking the conduction pathway [113]. Toxins, because of their peptide properties, such as rapid degradation, poor reversibility, and blood-brain barrier, are less than ideal for extended research use or drug development.

The next group of non-peptide BK_Ca_ channel inhibitors is a family of tremorgenic mycotoxins isolated from fungi and this group includes potent neurotoxin paxilline (PAX) [114]. Paxilline is the non-peptide neurotoxin most extensively used in research because of its high selectivity and reversibility of action, and capability of a 70% BK_Ca_ channel current inhibition at a concentration as low as 10 nM (K_i_ = 1.9 nM), and its site of action is located on the α-subunit and cytoplasmic side [115,116]. IbTX and PAX were used in multiple studies to determine the role of BK_Ca_ channels in the cardiovascular system. PAX resulted in a significant decrease (30%) in the heart rate of wild-type mice with no effect on mean blood pressure [57]. This effect was transient and concentration-dependent. To remove the possibility of systematic effects of PAX, isolated and perfused rat hearts also showed a decreased heart rate due to PAX (34%) and IbTX (60%) treatment [57]. IbTX is not cell permeable and those results suggested that BK_Ca_ channels expressed on the cell membrane of SAN cells play a role in heart rate regulation, not channels presented on IMM as we have seen in adult cardiomyocytes. Those findings were reproduced with a similar heart rate reduction caused by PAX, further supporting the presence of BK_Ca_ on the plasma membrane of SAN cells [11]. However, a recently published study conducted a rapid assessment of cardiac function after intravenous injection of PAX and IbTX [34]. On the one hand, PAX did produce significant bradycardia as reported [11,57], while, on the other hand, IbTX treatment showed no changes and PAX effects were reversible within 15 min after injection [34]. The contrasting findings (in vivo vs. ex vivo) with IbTX demonstrate the need for further investigation to determine the localization and biophysical properties of BK_Ca_ channels in SAN cells that are responsible for heart rate regulation. An in vivo implanted telemetry EKG monitoring system that allowed continuous monitoring in awake unrestrained mice after intraperitoneal injection of PAX showed a significant heart rate reduction, prolongation of the P-P interval, without prolongation of the P-R interval [11]. Those findings indicate that the mechanism involves SAN cells’ firing rate (spontaneous depolarization), with AV node conduction unaffected [11]. To exclude the autonomous effects of PAX, perforated patch-clamp recordings of isolated SAN cells were perfused with PAX, and seven out of eight cells showed a significant reduction of the firing rate consistent with in vivo findings [11]. Action potential (AP) waveform analysis showed a slowing of the diastolic depolarization without changes in the depolarization duration fitting, with the telemetric findings in vivo [11]. Like in the case of the activators, endogenous molecules play the role of BK_Ca_ channel blockers. Heme is a fundamentally important molecule and evidence suggests that free intracellular heme acts as a non-genomic signaling molecule that can acutely modulate BK_Ca_ channels [117,118]. Heme regulatory actions have been examined under a variety of physiological conditions, showing that it modifies the voltage-dependent gating by shifting the G-V curve towards more depolarizing voltages [119,120]. This regulation remained similar at saturating levels of divalent cations, showing that heme disrupts both Ca^2+^ and voltage-dependent gating, resulting in decreasing ionic currents. However, at more hyperpolarized and potentially more physiological voltages, heme actually enhanced channel activity [99,119]. Furthermore, CO reversed BK_Ca_ channel inhibition by heme, but not hemin (oxidized heme), and on BK_Ca_ binding, CO heme switches from being a channel inhibitor to an activator, making heme a functional CO receptor for the BK_Ca_ channels [121].

BK_Ca_ blockers were used in combination with activators to farther validate BK_Ca_ channels’ involvement in the area of interest that especially applies to I/R injury research. As we previously mentioned, BK_Ca_ was localized in IMM of cardiomyocytes, and treatment with NS1619 (BK_Ca_ opener) protected the heart during I/R injury, reflected in the significantly reduced infarct size [38]. This effect was reversed by PAX, which allowed the identification of BK_Ca_ channels as a major player in cardioprotection, and since then, those finding have been confirmed in numerous animal models [38,41,43,77,78,79,80,81,122,123]. For clarification of the mechanism by which BK_Ca_ channels resulted in a cardioprotective effect, close monitoring of mitochondrial changes during I/R injury was required. Continuous monitoring of NADH, superoxide, and mitochondrial Ca^2+^ levels in guinea pig hearts subjected to I/R injury revealed a significant reduction in mitochondrial Ca^2+^ capacity, ROS production, and levels of NADH on treatment with NS1619, which affected the end result: Infarct size was reduced by more than 50% in comparison to the control group [41]. Those cardioprotective effects were negated by PAX and superoxide dismutase, which indicated that both BK_Ca_ channels and superoxide activity are necessary to elicit cytoprotective effects [41]. At present, the proposed mechanism of cytoprotection starts with preconditioning, which leads to a mild increase of superoxide levels, which trigger the activation of BK_Ca_ channels. K^+^ influx into mitochondria partially depolarizes IMM and reduces Ca^2+^ influx during I/R injury, preventing mitochondrial Ca^2+^ overload, mPTP opening, and apoptosis initiation [41]. This proposed mechanism is being evaluated in genetic animal models, which we will review in the next section.

## 5. Genetic Animal Models

To further evaluate the role of BK_Ca_ channels in cardiac physiology and pathophysiology, several genetic mouse models were developed. The first reported *Kcnma1^-/-^* (*Kcnma1* global knockout) mice colony showed approximately normal Mendelian birth ratios of 28.5% for *Kcnma1^+/+^*, 50% for *Kcnma1^+/-^*, and 21.5% for *Kcnma1^-/-^* (296 mice) [124]. Similar to Slopoke flies [16], *Kcnma1^-/-^* animals were 27% smaller at 2 weeks than their littermates, but by week 5, null-mutant mice achieved a similar body weight [124]. *Kcnma1^-/-^* mice showed moderate ataxia and deficits in motor performance [124]. *Kcnma1^-/-^* were able to produce offspring, but the success rate was significantly reduced as only 1 out of 20 *Kcnma1^-/-^* males crossed with wild-type females was able to sire a normal-sized litter [124]. Additionally, 40% of *Kcnma1^-/-^* mice on the inbred FVB/NJ background died at 2.2 ± 0.3 months from unknown causes [124]. The use of *Kcnma1^-/-^* mice allowed researchers to further evaluate the specificity and side effects of BK_Ca_ channel agonists and blockers. BK_Ca_ channel blockers, Loritrem B, and PAX had no effects on heart rate and blood pressure in *Kcnma1^-/-^* mice, while administration of those inhibitors in conscious wild-type littermates caused a 30% and 42% reduction in heart rate, respectively [57]. This finding suggests that bradycardia was specifically mediated by α-subunit activity. Interestingly, the baseline heart rate did not vary between *Kcnma1^-/-^* and wild-type animals [57]. Lai et al. reported a similar basal heart rate in *Kcnma1^-/-^* and wild-type groups, confirming previous findings [11]. Analysis of the electrocardiogram (EKG) intervals in *Kcnma1^-/-^* mice showed no difference in P-P and R-R intervals when compared to wild-type mice [11]. Cells isolated from SAN of *Kcnma1^-/-^* mice showed slower basal firing rates and prolongation of the diastolic depolarization, mimicking the characteristics of wild-type SAN cells treated with PAX [11]. Those findings suggested that the unchanged basal heart rate in vivo is likely to be the extrinsic compensatory mechanism or *Kcnma1^-/-^* SAN cells compensate from the membrane potential and achieve homeostasis, while wild-type cells are unable to do so with acute inhibition of BK_Ca_ [11]. We determined mitoBK_Ca_ as a splice variant that requires the expression of the DEC exonic sequence at the c-terminus to target the inner mitochondrial membrane [5]. In the same study, NS1619-preconditioning cardioprotective effects were negated in *Kcnma1^-/-^* mice [5]. Soltysinska et al. used transmission electron microscopy to evaluate ventricular cardiomyocytes isolated from *Kcnma1^-/-^* mice and did not detect any abnormalities in the mitochondria structure and matrix dimensions [42]. In *Drosophila*, mitochondria in flight muscles showed cristae disintegration and swelling [16]. Surprisingly, oxidative phosphorylation capacities at normoxia and upon re-oxygenation after anoxia were significantly reduced in *Kcnma1^-/-^* cardiomyocytes [42] but not in flies during aging in the absence of BK_Ca_ channels [16]. Wild-type and *Kcnma1^-/-^* underwent ex vivo I/R injury with or without ischemic preconditioning [42]. Wild-type hearts showed a significant reduction in infarct size 28±3% (of the area at risk) when compared with *Kcnma1^-/-^* hearts 58 ± 5% (of the area at risk), with these findings suggesting that the BK_Ca_ channel mediates the cardioprotective benefits of ischemic preconditioning and supporting the conclusion from several other studies that the implicated BK_Ca_ channels in the respiratory chain and its disruption influence the infarct size [5,38,42,50].

The conditional mutant lacking the endogenous *Kcnma1* exclusively from cardiomyocytes (*CM-Kcnma1-Cre^fl/fl^*) brought a new level of specificity to the research of cardioprotective mechanisms by removing systemic effects that had to be taken under consideration in *Kcnma1^-/-^* global knockout. *CM-Kcnma1-Cre^fl/fl^* mice did not develop an obvious phenotype, exhibiting a normal body and heart weight; however, echocardiography showed a mild reduction in the cardiac ejection fraction, fraction shortening, heart rate, and significantly lower arterial blood pressure [47]. Animals were also subjected to an open-chest model of myocardial infarction and cardiac damage was compared between wild-type, *Kcnma1^-/-^* (global knockout), *CM-Kcnma1-Cre^fl/fl,^*, and *SM-Kcnma1-Cre^fl/fl^* (conditional mutant lacking *Kcnma1* from smooth muscle) [47]. Infarct size was significantly larger in *Kcnma1^-/-^* and *CM-Kcnma1-Cre^fl/f^* when compared with wild-type animals, revealing that the cardiomyocyte-specific BK_Ca_ channel accounts for >50% of the cardioprotection [47]. Infarct size in *SM-Kcnma1-Cre^fl/fl^* was similar to the wild-type group, showing that BK_Ca_ channels in vascular smooth muscle were not involved in pathological events elicited by I/R injury [47]. Furthermore, histological results were confirmed with serum cardiac troponin I levels, where larger infarct corresponded with higher troponin serum levels [47]. The administration of PAX resulted in a significant increase in heart damage in wild-type mice but had no effects on *CM-Kcnma1-Cre^fl/fl^* animals [47]. Interestingly, NS11021 treatment reduced infarct size in both wild-type and *CM-Kcnma1-Cre^fl/fl^*, although the effects were much smaller in knockout animals, which again brings back a question about NS11021 specificity that we discussed in the agonist section [47]. This study also tested the hypothesis that cardiomyocyte BK_Ca_ channels function within the cGMP/cGKI pathway with the use of guanylate cyclase activators (riociguat and cinaciguat). In wild-type mice, I/R with the administration of agents significantly reduced infarct size (42.5% riociguat, and 50.5% for cinaciguat) [47]. This cardioprotection was strongly attenuated in the case of riociguat and completely abolished in the case of cinaciguat and these results confirm that cGMP/cGKI pathway signaling contributes to cardiomyocyte protection during I/R injury [47]. This mechanism depends on BK_Ca_ channel activity, which agrees with previous studies where BK_Ca_ channels were directly stimulated by cGMP/cGKI [125,126,127]. These data indicate that mitoBK_Ca_ is possibly preset downstream of the cGMP pathway. To evaluate long-term changes, wild-type and *CM-Kcnma1-Cre^fl/fl^* were followed for 4 weeks post I/R injury. There was no difference in the survival rate, heart weight, or ventricular function as both groups showed similar results; however, levels of fibrosis were significantly increased in the *CM-Kcnma1-Cre^fl/fl^* group [47]. These studies present strong evidence that the cardioprotective effect depends on BK_Ca_ channel activity in cardiomyocytes, where BK_Ca_ is present in inner mitochondrial membranes.

Pharmacological activation implicated cardiomyocyte BK_Ca_ in cardioprotection; however, the usage of agonists remains controversial due to their non-specific effects. This limitation was addressed by using a genetic model that expressed a *Kcnma1*^R207Q^ constitutively active BK_Ca_ channel mutant (Tg-BK_Ca_^R207Q^) [46]. Tg-BK_Ca_^R207Q^ did not present any adverse cardiovascular phenotype, as the cardiac function parameters measured by echocardiography indicated no differences between wild-type and Tg-BK_Ca_^R207Q^ animals in the left ventricular ejection fraction, fraction shortening, and aortic velocity [46]. Hearts from wild-type and Tg-BK_Ca_^R207Q^ were subjects of I/R injury, with or without ischemic preconditioning. Results showed that in both models, with or without ischemic preconditioning, infarct size was significantly smaller in the Tg-BK_Ca_^R207Q^ group, showing that Tg-BK_Ca_^R207Q^ was better protected from I/R injury than the wild type [46]. Since ROS production is implicated in cardioprotection, ROS was measured in the heart-isolated mitochondria of Tg-BK_Ca_^R207Q^ mice. Genetic activation of BK_Ca_ channels reduced ROS after I/R stress and preconditioning further decreased it [46], directly implicating BK_Ca_ channels in the modulation of mitochondrial ROS generation. Those results showed that activation of the BK_Ca_ channel is essential for cardiac recovery from I/R injury and is a factor in ischemic preconditioning-mediated cardioprotection.

## 6. BK_Ca_ as a Therapeutic Target for Cardioprotection

Data from 2017 show that 48% (121.5 million) of adults in the US suffer from cardiovascular disease and it is a leading cause of death (30.5% of deaths in 2017 in the US) [128]. Coronary heart disease, including AMI, was attributed to 42.6% of cardiovascular disease-related deaths, followed by stroke (17.0%) and high blood pressure (10.5%) [128]. AMI in addition to acute damage can develop into heart failure, which is another major factor in cardiovascular mortality [128]. Cardiomyocyte death during AMI develops in two stages, ischemic injury and reperfusion injury, which contributes up to 50% of infarct size, but clinically, reopening the occluded vessel is the best AMI treatment available at this moment [129]. Infarct size is a determinant of myocardial functional recovery and therapies aimed at the reduction of reperfusion injuries are highly desired.

The first preconditioning intervention was reported in 1986 when it was demonstrated that four short ischemic-reperfusion pulses resulted in a dramatic 75% decrease in infarct size [130]. The nature of this protective mechanism suggested the involvement of the K^+^ channel. The role of BK_Ca_ channels in cardiomyocytes was neglected because of their absence from the plasma membrane of cardiomyocytes [10]. However, in 2002, O’Rourke’s group reported voltage- and calcium-dependent potassium currents on mitoplasts isolated from guinea pig cardiomyocytes [38]. The recorded currents had a single-channel conductance of ~307 pS and were blocked by ChTX [38]. Furthermore, NS1619 protected the heart from the ischemic event and this effect was blocked by PAX [38]. Subsequent studies confirmed that NS1619 protected the heart from I/R injury in a number of animal species and administration of NS11021, before or right after, also protected the heart from I/R injury [50,131]. Because most of the data addressing the role of mitoBK_Ca_ channels in cardioprotection relied on pharmacological tools, the data published were questioned because both activators and blockers displayed unspecific effects. Our group resolved controversies about pharmacology, showing that the cardioprotection offered by NS1619 was lost in *Kcnma1^-/-^* mice and clearly demonstrating BK_Ca_ channels’ involvement in cardioprotection, which was also confirmed with the use of cardiomyocyte-specific knockouts, *CM-Kcnma1-Cre^fl/fl^* [5,47]. Additionally, changes in ROS production and attenuated oxidative phosphorylation in *Kcnma1^-/-^* cardiomyocytes were also observed, suggesting the role of mitoBK_Ca_ in fine-tuning the oxidative state of the cell [42]. We still do not have a clear picture of how the BK_Ca_ channel contributes to cardioprotection, although a few mechanisms have been proposed. The BK_Ca_ channel affects mitochondrial Ca^2+^ accumulation, K^+^ influx leads to partial depolarization if the IMM reduces the driving force for Ca^2+^ entry, and prevents Ca^2+^ overload during I/R injury [41,44]. Additionally, pre-treatment with NS1619 increased mitochondria Ca^2+^ retention capacity, an effect that was lost in *Kcnma1^-/-^* mice [5].

The role of mitoBK_Ca_ has also been shown in the regulation of ROS production in various models. Isolated mitochondria from cardiomyocytes and neurons showed decreased ROS production when stimulated with NS1619, which was confirmed in isolated hearts and a *Kcnma1^-/-^* mice model, where mitochondria isolated from knockout mice showed elevated ROS production after the anoxic event [41,42,131]. Moreover, mitoBK_Ca_ opening has been shown to improve mitochondrial energy production by swelling of the mitochondrial matrix [61]. An attenuated phosphorylation capacity was also identified in a *CM-Kcnma1-Cre^fl/fl^* mice model, which further connected mitoBK_Ca_ activity with ATP preservation [42]. During reperfusion, after an ischemic event, mitoBK_Ca_ activity would improve oxidative phosphorylation, decrease ROS production, and improve mitochondria Ca^2+^ retention, preventing opening of the mitochondrial permeability transition pore (mPTP), which would lead to mitochondria collapse, termination of ATP synthesis, and cardiomyocyte death [40]. The recent findings with the use of pharmacological tools and genetic models have clearly demonstrated that BK_Ca_ channels are important for cardioprotection, regulation of vascular tone, and mitochondrial function. However, there are still variations in results that seem to depend on the methods chosen and experimental design, and imperfect activators and blockers that display a number of unspecific side effects. The number of synthetic BK_Ca_ activators have been developed through the years by different pharmaceutical companies, with promising results in animal models, but with limited success in clinical trials [131]. The research established mitoBK_Ca_ channels as a promising target for limiting reperfusion damage and correcting long-term events that occur after AMI, but further research will be needed to develop clinical pharmacological tools for cardiac disease in the future.

## 7. Conclusions and Future Directions

Over the past 30 years, BK_Ca_ has been cloned and characterized in several pathological and physiological conditions. The establishment of the KCNMA1-channelopathy consortium has truly pushed BK_Ca_ as a therapeutic target for several neuronal and other possible diseases. In the heart, several animal models have shown that activation of BK_Ca_ channels, either pharmacologically or genetically, protects the heart from ischemia-reperfusion injury. In humans, the *Kcnma1* gene is positioned within the first locus mapped from familial atrial fibrillation on chr10q23. Genetic-linkage analysis indicated that the gene responsible for atrial fibrillation (AF) is located on chromosome 10q in the region of 10q22–q24 [36]. The original candidate genes thought to be involved in AF were β-adrenergic receptor (ADRB1)13 and α-adrenergic receptor (ADRA2)14 located on 10q23–q26, and G-protein-coupled receptor kinase (GPRK5) 15, which interacts with adrenergic receptors. Sequence analysis of these candidate genes failed to identify any pathogenic variants. The chromosome 10q22-q24 AF locus also overlaps with loci mapped with other cardiac disorders, such as arrhythmogenic right ventricular cardiomyopathy, dilated cardiomyopathy, and hypoplastic left heart syndrome. *Kcnma1* has largely been ignored from screens as it was originally reported to be absent in cardiomyocytes [35]. Given the recent studies focused on establishing their molecular identity, its roles in the regulation of heart rate, and its roles of cardiomyocyte BK_Ca_ channels in cardioprotection, and its location in Chr10q23, it is a very promising candidate gene for cardiac pathophysiology.

## References

[1] Latorre R., Vergara C., Hidalgo C. (1982). Reconstitution in planar lipid bilayers of a Ca2+-dependent K+ channel from transverse tubule membranes isolated from rabbit skeletal muscle. Proc. Natl. Acad. Sci. USA.

[2] Pallotta B.S., Magleby K.L., Barrett J.N. (1981). Single channel recordings of Ca2+-activated K+ currents in rat muscle cell culture. Nature.

[3] Marty A. (1981). Ca-dependent K channels with large unitary conductance in chromaffin cell membranes. Nature.

[4] Toro L., Li M., Zhang Z., Singh H., Wu Y., Stefani E. (2014). MaxiK channel and cell signalling. Pflügers Arch. Eur. J. Physiol..

[5] Singh H., Lu R., Bopassa J.C., Meredith A.L., Stefani E., Toro L. (2013). MitoBK(Ca) is encoded by the Kcnma1 gene, and a splicing sequence defines its mitochondrial location. Proc. Natl. Acad. Sci. USA.

[6] Li B., Jie W., Huang L., Wei P., Li S., Luo Z., Friedman A.K., Meredith A.L., Han M.H., Zhu X.H. (2014). Nuclear BK channels regulate gene expression via the control of nuclear calcium signaling. Nat. Neurosci..

[7] Kaufman R.J. (1999). Stress signaling from the lumen of the endoplasmic reticulum: Coordination of gene transcriptional and translational controls. Genes. Dev..

[8] Jing G., Wang J.J., Zhang S.X. (2012). ER stress and apoptosis: A new mechanism for retinal cell death. Exp. Diabetes. Res..

[9] Cao Q., Zhong X.Z., Zou Y., Zhang Z., Toro L., Dong X.P. (2015). BK Channels Alleviate Lysosomal Storage Diseases by Providing Positive Feedback Regulation of Lysosomal Ca2+ Release. Dev. Cell.

[10] Singh H., Stefani E., Toro L. (2012). Intracellular BK(Ca) (iBK(Ca)) channels. J. Physiol..

[11] Lai M.H., Wu Y., Gao Z., Anderson M.E., Dalziel J.E., Meredith A.L. (2014). BK channels regulate sinoatrial node firing rate and cardiac pacing in vivo. Am. J. Physiol. Heart Circ. Physiol..

[12] Lifshitz L.M., Carmichael J.D., Lai F.A., Sorrentino V., Bellve K., Fogarty K.E., ZhuGe R. (2011). Spatial organization of RYRs and BK channels underlying the activation of STOCs by Ca(2+) sparks in airway myocytes. J. Gen. Physiol..

[13] Meredith A.L., Wiler S.W., Miller B.H., Takahashi J.S., Fodor A.A., Ruby N.F., Aldrich R.W. (2006). BK calcium-activated potassium channels regulate circadian behavioral rhythms and pacemaker output. Nat. Neurosci..

[14] Werner M.E., Zvara P., Meredith A.L., Aldrich R.W., Nelson M.T. (2005). Erectile dysfunction in mice lacking the large-conductance calcium-activated potassium (BK) channel. J. Physiol..

[15] Heppner T.J., Bonev A.D., Nelson M.T. (1997). Ca(2+)-activated K+ channels regulate action potential repolarization in urinary bladder smooth muscle. Am. J. Physiol..

[16] Rao S.G., Bednarczyk P., Towheed A., Shah K., Karekar P., Ponnalagu D., Jensen H.N., Addya S., Reyes B.A.S., van Bockstaele E.J. (2019). BKCa (Slo) Channel Regulates Mitochondrial Function and Lifespan in Drosophila melanogaster. Cells.

[17] Bailey C.S., Moldenhauer H.J., Park S.M., Keros S., Meredith A.L. (2019). KCNMA1-linked channelopathy. J. Gen. Physiol..

[18] Du X., Carvalho-de-Souza J.L., Wei C., Carrasquel-Ursulaez W., Lorenzo Y., Gonzalez N., Kubota T., Staisch J., Hain T., Petrossian N. (2020). Loss-of-function BK channel mutation causes impaired mitochondria and progressive cerebellar ataxia. Proc. Natl. Acad. Sci. USA.

[19] Kcnma1 Channelopathy International Advocacy Foundation. https://www.kciaf.org/.

[20] Magleby K.L. (2003). Gating mechanism of BK (Slo1) channels: So near, yet so far. J. Gen. Physiol..

[21] Horrigan F.T., Aldrich R.W. (2002). Coupling between voltage sensor activation, Ca2+ binding and channel opening in large conductance (BK) potassium channels. J. Gen. Physiol..

[22] Stefani E., Ottolia M., Noceti F., Olcese R., Wallner M., Latorre R., Toro L. (1997). Voltage-controlled gating in a large conductance Ca2+-sensitive K+channel (hslo). Proc. Natl. Acad. Sci. USA.

[23] Meera P., Wallner M., Song M., Toro L. (1997). Large conductance voltage- and calcium-dependent K+ channel, a distinct member of voltage-dependent ion channels with seven N-terminal transmembrane segments (S0-S6), an extracellular N terminus, and an intracellular (S9-S10) C terminus. Proc. Natl. Acad. Sci. USA.

[24] Yuan P., Leonetti M.D., Pico A.R., Hsiung Y., MacKinnon R. (2010). Structure of the human BK channel Ca2+-activation apparatus at 3.0 A resolution. Science.

[25] Cox D.H., Aldrich R.W. (2000). Role of the beta1 subunit in large-conductance Ca(2+)-activated K(+) channel gating energetics. Mechanisms of enhanced Ca(2+) sensitivity. J. Gen. Physiol..

[26] Li Q., Yan J. (2016). Modulation of BK Channel Function by Auxiliary Beta and Gamma Subunits. Int. Rev. Neurobiol..

[27] Gonzalez-Perez V., Lingle C.J. (2019). Regulation of BK Channels by Beta and Gamma Subunits. Annu. Rev. Physiol..

[28] Lam A., Karekar P., Shah K., Hariharan G., Fleyshman M., Kaur H., Singh H., Rao S.G. (2018). Drosophila Voltage-Gated Calcium Channel alpha1-Subunits Regulate Cardiac Function in the Aging Heart. Sci. Rep..

[29] Ponnalagu D., Singh H. (2020). Insights into the Role of Mitochondrial Ion Channels in Inflammatory Response. Front. Physiol..

[30] Ponnalagu D., Singh H. (2017). Anion Channels of Mitochondria. Handb. Exp. Pharmacol..

[31] Ponnalagu D., Rao S.G., Farber J., Xin W., Hussain A.T., Shah K., Tanda S., Berryman M., Edwards J.C., Singh H. (2016). Molecular identity of cardiac mitochondrial chloride intracellular channel proteins. Mitochondrion.

[32] Martin L.J., Lau E., Singh H., Vergnes L., Tarling E.J., Mehrabian M., Mungrue I., Xiao S., Shih D., Castellani L. (2012). ABCC6 localizes to the mitochondria-associated membrane. Circ. Res..

[33] Rao S.G., Patel N.J., Singh H. (2020). Intracellular Chloride Channels: Novel Biomarkers in Diseases. Front. Physiol..

[34] Patel N.H., Johannesen J., Shah K., Goswami S.K., Patel N.J., Ponnalagu D., Kohut A.R., Singh H. (2018). Inhibition of BKCa negatively alters cardiovascular function. Physiol. Rep..

[35] Koster O.F., Szigeti G.P., Beuckelmann D.J. (1999). Characterization of a [Ca2+]i-dependent current in human atrial and ventricular cardiomyocytes in the absence of Na+ and K+. Cardiovasc. Res..

[36] Brugada R., Tapscott T., Czernuszewicz G.Z., Marian A.J., Iglesias A., Mont L., Brugada J., Girona J., Domingo A., Bachinski L.L. (1997). Identification of a genetic locus for familial atrial fibrillation. N. Engl. J. Med..

[37] Dopico A.M., Bukiya A.N., Jaggar J.H. (2018). Calcium- and voltage-gated BK channels in vascular smooth muscle. Pflugers Arch. Eur. J. Physiol..

[38] Xu W., Liu Y., Wang S., McDonald T., van Eyk J.E., Sidor A., O’Rourke B. (2002). Cytoprotective role of Ca2+- activated K+ channels in the cardiac inner mitochondrial membrane. Science.

[39] Wojtovich A.P., Nadtochiy S.M., Urciuoli W.R., Smith C.O., Grunnet M., Nehrke K., Brookes P.S. (2013). A non-cardiomyocyte autonomous mechanism of cardioprotection involving the SLO1 BK channel. PeerJ.

[40] Szabo I., Zoratti M. (2014). Mitochondrial channels: Ion fluxes and more. Physiol. Rev..

[41] Stowe D.F., Aldakkak M., Camara A.K., Riess M.L., Heinen A., Varadarajan S.G., Jiang M.T. (2006). Cardiac mitochondrial preconditioning by Big Ca2+-sensitive K+ channel opening requires superoxide radical generation. Am. J. Physiol. Heart Circ. Physiol..

[42] Soltysinska E., Bentzen B.H., Barthmes M., Hattel H., Thrush A.B., Harper M.E., Qvortrup K., Larsen F.J., Schiffer T.A., Losa-Reyna J. (2014). KCNMA1 encoded cardiac BK channels afford protection against ischemia-reperfusion injury. PLoS ONE.

[43] Shi Y., Jiang M.T., Su J., Hutchins W., Konorev E., Baker J.E. (2007). Mitochondrial big conductance KCa channel and cardioprotection in infant rabbit heart. J. Cardiovasc. Pharmacol..

[44] Sato T., Saito T., Saegusa N., Nakaya H. (2005). Mitochondrial Ca2+-activated K+ channels in cardiac myocytes: A mechanism of the cardioprotective effect and modulation by protein kinase A. Circulation.

[45] Ruttiger L., Sausbier M., Zimmermann U., Winter H., Braig C., Engel J., Knirsch M., Arntz C., Langer P., Hirt B. (2004). Deletion of the Ca2+-activated potassium (BK) alpha-subunit but not the BKbeta1-subunit leads to progressive hearing loss. Proc. Natl. Acad. Sci. USA.

[46] Goswami S.K., Ponnalagu D., Hussain A.T., Shah K., Karekar P., Rao S.G., Meredith A.L., Khan M., Singh H. (2018). Expression and Activation of BKCa Channels in Mice Protects Against Ischemia-Reperfusion Injury of Isolated Hearts by Modulating Mitochondrial Function. Front. Cardiovasc. Med..

[47] Frankenreiter S., Bednarczyk P., Kniess A., Bork N.I., Straubinger J., Koprowski P., Wrzosek A., Mohr E., Logan A., Murphy M.P. (2017). cGMP-Elevating Compounds and Ischemic Conditioning Provide Cardioprotection Against Ischemia and Reperfusion Injury via Cardiomyocyte-Specific BK Channels. Circulation.

[48] Brenner R., Perez G.J., Bonev A.D., Eckman D.M., Kosek J.C., Wiler S.W., Patterson A.J., Nelson M.T., Aldrich R.W. (2000). Vasoregulation by the beta1 subunit of the calcium-activated potassium channel. Nature.

[49] Brayden J.E., Nelson M.T. (1992). Regulation of arterial tone by activation of calcium-dependent potassium channels. Science.

[50] Bentzen B.H., Osadchii O., Jespersen T., Hansen R.S., Olesen S.P., Grunnet M. (2009). Activation of big conductance Ca(2+)-activated K (+) channels (BK) protects the heart against ischemia-reperfusion injury. Pflugers Arch..

[51] Balderas E., Zhang J., Stefani E., Toro L. (2015). Mitochondrial BKCa channel. Front. Physiol..

[52] Gollasch M., Ried C., Bychkov R., Luft F.C., Haller H. (1996). K+ currents in human coronary artery vascular smooth muscle cells. Circ. Res..

[53] Leblanc N., Wan X., Leung P.M. (1994). Physiological role of Ca(2+)-activated and voltage-dependent K+ currents in rabbit coronary myocytes. Am. J. Physiol..

[54] Nelson M.T., Cheng H., Rubart M., Santana L.F., Bonev A.D., Knot H.J., Lederer W.J. (1995). Relaxation of arterial smooth muscle by calcium sparks. Science.

[55] McManus O.B., Helms L.M., Pallanck L., Ganetzky B., Swanson R., Leonard R.J. (1995). Functional role of the beta subunit of high conductance calcium-activated potassium channels. Neuron.

[56] Grimm P.R., Sansom S.C. (2010). BK channels and a new form of hypertension. Kidney Int..

[57] Imlach W.L., Finch S.C., Miller J.H., Meredith A.L., Dalziel J.E. (2010). A role for BK channels in heart rate regulation in rodents. PLoS ONE.

[58] Mangoni M.E., Nargeot J. (2008). Genesis and regulation of the heart automaticity. Physiol. Rev..

[59] Vandael D.H., Marcantoni A., Mahapatra S., Caro A., Ruth P., Zuccotti A., Knipper M., Carbone E. (2010). Ca(v)1.3 and BK channels for timing and regulating cell firing. Mol. Neurobiol..

[60] Fakler B., Adelman J.P. (2008). Control of K(Ca) channels by calcium nano/microdomains. Neuron.

[61] Aon M.A., Cortassa S., Wei A.C., Grunnet M., O’Rourke B. (2010). Energetic performance is improved by specific activation of K+ fluxes through K(Ca) channels in heart mitochondria. Biochim. Biophys. Acta..

[62] Xu C., Chen B., Wang W., Tian Y., Zhao H., Jiang B., Gao B., Qin S., Yue M., Qi G. (2000). Detecting residual ischemia and identifying coronary artery disease after myocardial infarction using dobutamine technetium-99m-MIBI SPECT. Chin. Med. J..

[63] Tang Q.Y., Qi Z., Naruse K., Sokabe M. (2003). Characterization of a functionally expressed stretch-activated BKca channel cloned from chick ventricular myocytes. J. Membr. Biol..

[64] Zhao H., Yu Y., Wu X., Liu S., Liu B., Du J., Li B., Jiang L., Feng X. (2017). A Role of BK Channel in Regulation of Ca(2+) Channel in Ventricular Myocytes by Substrate Stiffness. Biophys. J..

[65] Zhao H.C., Agula H., Zhang W., Wang F., Sokabe M., Li L.M. (2010). Membrane stretch and cytoplasmic Ca2+ independently modulate stretch-activated BK channel activity. J. Biomech..

[66] Li H., Xu J., Shen Z.S., Wang G.M., Tang M., Du X.R., Lv Y.T., Wang J.J., Zhang F.F., Qi Z. (2019). The neuropeptide GsMTx4 inhibits a mechanosensitive BK channel through the voltage-dependent modification specific to mechano-gating. J. Biol. Chem..

[67] Banerjee I., Fuseler J.W., Price R.L., Borg T.K., Baudino T.A. (2007). Determination of cell types and numbers during cardiac development in the neonatal and adult rat and mouse. Am. J. Physiol. Heart Circ. Physiol..

[68] Li G.R., Sun H.Y., Chen J.B., Zhou Y., Tse H.F., Lau C.P. (2009). Characterization of multiple ion channels in cultured human cardiac fibroblasts. PLoS ONE.

[69] Wang Y.J., Sung R.J., Lin M.W., Wu S.N. (2006). Contribution of BK(Ca)-channel activity in human cardiac fibroblasts to electrical coupling of cardiomyocytes-fibroblasts. J. Membr. Biol..

[70] Wang Y.J., Lin M.W., Wu S.N., Sung R.J. (2007). The activation by estrogen receptor agonists of the BK(Ca)-channel in human cardiac fibroblasts. Biochem. Pharmacol..

[71] Chilton L., Ohya S., Freed D., George E., Drobic V., Shibukawa Y., Maccannell K.A., Imaizumi Y., Clark R.B., Dixon I.M. (2005). K+ currents regulate the resting membrane potential, proliferation, and contractile responses in ventricular fibroblasts and myofibroblasts. Am. J. Physiol. Heart Circ. Physiol..

[72] Vasquez C., Mohandas P., Louie K.L., Benamer N., Bapat A.C., Morley G.E. (2010). Enhanced fibroblast-myocyte interactions in response to cardiac injury. Circ. Res..

[73] Cui J., Yang H., Lee U.S. (2009). Molecular mechanisms of BK channel activation. Cell Mol. Life Sci..

[74] Augustynek B., Kunz W.S., Szewczyk A. (2017). Guide to the Pharmacology of Mitochondrial Potassium Channels. Handb. Exp. Pharmacol..

[75] Olesen S.P., Munch E., Watjen F., Drejer J. (1994). NS 004--an activator of Ca(2+)-dependent K+ channels in cerebellar granule cells. Neuroreport.

[76] Olesen S.P., Munch E., Moldt P., Drejer J. (1994). Selective activation of Ca(2+)-dependent K+ channels by novel benzimidazolone. Eur. J. Pharmacol..

[77] Redel A., Lange M., Jazbutyte V., Lotz C., Smul T.M., Roewer N., Kehl F. (2008). Activation of mitochondrial large-conductance calcium-activated K+ channels via protein kinase A mediates desflurane-induced preconditioning. Anesth. Analg..

[78] Wang X., Yin C., Xi L., Kukreja R.C. (2004). Opening of Ca2+-activated K+ channels triggers early and delayed preconditioning against I/R injury independent of NOS in mice. Am. J. Physiol. Heart Circ. Physiol..

[79] Cao C.M., Xia Q., Gao Q., Chen M., Wong T.M. (2005). Calcium-activated potassium channel triggers cardioprotection of ischemic preconditioning. J. Pharmacol. Exp. Ther..

[80] Gao Q., Zhang S.Z., Cao C.M., Bruce I.C., Xia Q. (2005). The mitochondrial permeability transition pore and the Ca2+-activated K+ channel contribute to the cardioprotection conferred by tumor necrosis factor-alpha. Cytokine.

[81] Shintani Y., Node K., Asanuma H., Sanada S., Takashima S., Asano Y., Liao Y., Fujita M., Hirata A., Shinozaki Y. (2004). Opening of Ca2+-activated K+ channels is involved in ischemic preconditioning in canine hearts. J. Mol. Cell Cardiol..

[82] Park W.S., Kang S.H., Son Y.K., Kim N., Ko J.H., Kim H.K., Ko E.A., Kim C.D., Han J. (2007). The mitochondrial Ca2+-activated K+ channel activator, NS 1619 inhibits L-type Ca2+ channels in rat ventricular myocytes. Biochem. Biophys. Res. Commun..

[83] Saleh S.N., Angermann J.E., Sones W.R., Leblanc N., Greenwood I.A. (2007). Stimulation of Ca2+-gated Cl- currents by the calcium-dependent K+ channel modulators NS1619 [1,3-dihydro-1-[2-hydroxy-5-(trifluoromethyl)phenyl]-5-(trifluoromethyl)-2H-benzi midazol-2-one] and isopimaric acid. J. Pharmacol. Exp. Ther..

[84] Edwards G., Niederste-Hollenberg A., Schneider J., Noack T., Weston A.H. (1994). Ion channel modulation by NS 1619, the putative BKCa channel opener, in vascular smooth muscle. Br. J. Pharmacol..

[85] Holland M., Langton P.D., Standen N.B., Boyle J.P. (1996). Effects of the BKCa channel activator, NS1619, on rat cerebral artery smooth muscle. Br. J. Pharmacol..

[86] Lukasiak A., Skup A., Chlopicki S., Lomnicka M., Kaczara P., Proniewski B., Szewczyk A., Wrzosek A. (2016). SERCA, complex I of the respiratory chain and ATP-synthase inhibition are involved in pleiotropic effects of NS1619 on endothelial cells. Eur. J. Pharmacol..

[87] Cancherini D.V., Queliconi B.B., Kowaltowski A.J. (2007). Pharmacological and physiological stimuli do not promote Ca(2+)-sensitive K+ channel activity in isolated heart mitochondria. Cardiovasc. Res..

[88] Bentzen B.H., Nardi A., Calloe K., Madsen L.S., Olesen S.P., Grunnet M. (2007). The small molecule NS11021 is a potent and specific activator of Ca2+-activated big-conductance K+ channels. Mol. Pharmacol..

[89] Cheng Y., Gu X.Q., Bednarczyk P., Wiedemann F.R., Haddad G.G., Siemen D. (2008). Hypoxia increases activity of the BK-channel in the inner mitochondrial membrane and reduces activity of the permeability transition pore. Cell Physiol. Biochem..

[90] Hermann A., Sitdikova G.F., Weiger T.M. (2015). Oxidative Stress and Maxi Calcium-Activated Potassium (BK) Channels. Biomolecules.

[91] Hou S., Xu R., Heinemann S.H., Hoshi T. (2008). The RCK1 high-affinity Ca2+ sensor confers carbon monoxide sensitivity to Slo1 BK channels. Proc. Natl. Acad. Sci. USA.

[92] Hou S., Heinemann S.H., Hoshi T. (2009). Modulation of BKCa channel gating by endogenous signaling molecules. Physiol. Bethesda.

[93] Hayabuchi Y., Nakaya Y., Matsuoka S., Kuroda Y. (1998). Effect of acidosis on Ca2+-activated K+ channels in cultured porcine coronary artery smooth muscle cells. Pflugers Arch..

[94] Hou S., Xu R., Heinemann S.H., Hoshi T. (2008). Reciprocal regulation of the Ca2+ and H+ sensitivity in the SLO1 BK channel conferred by the RCK1 domain. Nat. Struct. Mol. Biol..

[95] Lipton P. (1999). Ischemic cell death in brain neurons. Physiol. Rev..

[96] Avdonin V., Tang X.D., Hoshi T. (2003). Stimulatory action of internal protons on Slo1 BK channels. Biophys. J..

[97] Bolotina V.M., Najibi S., Palacino J.J., Pagano P.J., Cohen R.A. (1994). Nitric oxide directly activates calcium-dependent potassium channels in vascular smooth muscle. Nature.

[98] Brakemeier S., Eichler I., Knorr A., Fassheber T., Kohler R., Hoyer J. (2003). Modulation of Ca2+-activated K+ channel in renal artery endothelium in situ by nitric oxide and reactive oxygen species. Kidney Int..

[99] Li L., Rose P., Moore P.K. (2011). Hydrogen sulfide and cell signaling. Annu. Rev. Pharmacol. Toxicol..

[100] Peers C., Bauer C.C., Boyle J.P., Scragg J.L., Dallas M.L. (2010). Modulation of ion channels by hydrogen sulfide. Antioxid. Redox Signal..

[101] Telezhkin V., Brazier S.P., Cayzac S.H., Wilkinson W.J., Riccardi D., Kemp P.J. (2010). Mechanism of inhibition by hydrogen sulfide of native and recombinant BKCa channels. Respir. Physiol. Neurobiol..

[102] Santarelli L.C., Chen J., Heinemann S.H., Hoshi T. (2004). The beta1 subunit enhances oxidative regulation of large-conductance calcium-activated K+ channels. J. Gen. Physiol..

[103] Sitdikova G.F., Fuchs R., Kainz V., Weiger T.M., Hermann A. (2014). Phosphorylation of BK channels modulates the sensitivity to hydrogen sulfide (H2S). Front. Physiol..

[104] Borchert G.H., Hlavackova M., Kolar F. (2013). Pharmacological activation of mitochondrial BK(Ca) channels protects isolated cardiomyocytes against simulated reperfusion-induced injury. Exp. Biol. Med..

[105] Gutterman D.D., Miura H., Liu Y. (2005). Redox modulation of vascular tone: Focus of potassium channel mechanisms of dilation. Arterioscler. Thromb. Vasc. Biol..

[106] Yellen G. (1984). Ionic permeation and blockade in Ca2+-activated K+ channels of bovine chromaffin cells. J. Gen. Physiol..

[107] Iwatsuki N., Petersen O.H. (1985). Action of tetraethylammonium on calcium-activated potassium channels in pig pancreatic acinar cells studied by patch-clamp single-channel and whole-cell current recording. J. Membr. Biol..

[108] Lenaeus M.J., Vamvouka M., Focia P.J., Gross A. (2005). Structural basis of TEA blockade in a model potassium channel. Nat. Struct. Mol. Biol..

[109] Nardi A., Olesen S.P. (2008). BK channel modulators: A comprehensive overview. Curr. Med. Chem..

[110] Miller C., Moczydlowski E., Latorre R., Phillips M. (1985). Charybdotoxin, a protein inhibitor of single Ca2+-activated K+ channels from mammalian skeletal muscle. Nature.

[111] Panyi G., Possani L.D., de la Vega R.C.R., Gaspar R., Varga Z. (2006). K+ channel blockers: Novel tools to inhibit T cell activation leading to specific immunosuppression. Curr. Pharm. Des..

[112] Galvez A., Gimenez-Gallego G., Reuben J.P., Roy-Contancin L., Feigenbaum P., Kaczorowski G.J., Garcia M.L. (1990). Purification and characterization of a unique, potent, peptidyl probe for the high conductance calcium-activated potassium channel from venom of the scorpion Buthus tamulus. J. Biol. Chem..

[113] Candia S., Garcia M.L., Latorre R. (1992). Mode of action of iberiotoxin, a potent blocker of the large conductance Ca(2+)-activated K+ channel. Biophys. J..

[114] Nardi A., Calderone V., Chericoni S., Morelli I. (2003). Natural modulators of large-conductance calcium-activated potassium channels. Planta Med..

[115] Knaus H.G., McManus O.B., Lee S.H., Schmalhofer W.A., Garcia-Calvo M., Helms L.M., Sanchez M., Giangiacomo K., Reuben J.P., Smith A.B. (1994). Tremorgenic indole alkaloids potently inhibit smooth muscle high-conductance calcium-activated potassium channels. Biochemistry.

[116] Sanchez M., McManus O.B. (1996). Paxilline inhibition of the alpha-subunit of the high-conductance calcium-activated potassium channel. Neuropharmacology.

[117] Padmanaban G., Venkateswar V., Rangarajan P.N. (1989). Haem as a multifunctional regulator. Trends Biochem. Sci..

[118] Hou S., Reynolds M.F., Horrigan F.T., Heinemann S.H., Hoshi T. (2006). Reversible binding of heme to proteins in cellular signal transduction. Acc. Chem. Res..

[119] Horrigan F.T., Heinemann S.H., Hoshi T. (2005). Heme regulates allosteric activation of the Slo1 BK channel. J. Gen. Physiol..

[120] Tang X.D., Xu R., Reynolds M.F., Garcia M.L., Heinemann S.H., Hoshi T. (2003). Haem can bind to and inhibit mammalian calcium-dependent Slo1 BK channels. Nature.

[121] Jaggar J.H., Li A., Parfenova H., Liu J., Umstot E.S., Dopico A.M., Leffler C.W. (2005). Heme is a carbon monoxide receptor for large-conductance Ca2+-activated K+ channels. Circ. Res..

[122] Wang B., Jaffe D.B., Brenner R. (2014). Current understanding of iberiotoxin-resistant BK channels in the nervous system. Front. Physiol..

[123] Heinen A., Winning A., Schlack W., Hollmann M.W., Preckel B., Frassdorf J., Weber N.C. (2008). The regulation of mitochondrial respiration by opening of mKCa channels is age-dependent. Eur. J. Pharmacol..

[124] Meredith A.L., Thorneloe K.S., Werner M.E., Nelson M.T., Aldrich R.W. (2004). Overactive bladder and incontinence in the absence of the BK large conductance Ca2+-activated K+ channel. J. Biol. Chem..

[125] Kyle B.D., Hurst S., Swayze R.D., Sheng J., Braun A.P. (2013). Specific phosphorylation sites underlie the stimulation of a large conductance, Ca(2+)-activated K(+) channel by cGMP-dependent protein kinase. FASEB J..

[126] Swayze R.D., Braun A.P. (2001). A catalytically inactive mutant of type I cGMP-dependent protein kinase prevents enhancement of large conductance, calcium-sensitive K+ channels by sodium nitroprusside and cGMP. J. Biol. Chem..

[127] Zhou X.B., Wulfsen I., Utku E., Sausbier U., Sausbier M., Wieland T., Ruth P., Korth M. (2010). Dual role of protein kinase C on BK channel regulation. Proc. Natl. Acad. Sci. USA.

[128] Virani S.S., Alonso A., Benjamin E.J., Bittencourt M.S., Callaway C.W., Carson A.P., Chamberlain A.M., Chang A.R., Cheng S., Delling F.N. (2020). American Heart Association Council on, C. Prevention Statistics, and S. Stroke Statistics, Heart Disease and Stroke Statistics-2020 Update: A Report From the American Heart Association. Circulation.

[129] Hausenloy D.J., Yellon D.M. (2013). Myocardial ischemia-reperfusion injury: A neglected therapeutic target. J. Clin. Investig..

[130] Murry C.E., Jennings R.B., Reimer K.A. (1986). Preconditioning with ischemia: A delay of lethal cell injury in ischemic myocardium. Circulation.

[131] Bentzen B.H., Olesen S.P., Ronn L.C., Grunnet M. (2014). BK channel activators and their therapeutic perspectives. Front. Physiol..

